# Sociodemographic Factors Influencing Vietnamese Patient Satisfaction with Healthcare Services and Some Meaningful Empirical Thresholds

**Published:** 2018-01

**Authors:** Quan-Hoang VUONG

**Affiliations:** Western University Hanoi, Center for Interdisciplinary Social Research, Hanoi, Vietnam

**Keywords:** Health insurance, Threshold, Medical expenditures, Healthcare policy, Vietnam

## Abstract

**Background:**

This short communication report some new results obtained from a medical survey among 900 Vietnamese patients in 2015, looking into possibly influential sociodemographic factors as far as patient satisfaction is concerned, to establish empirical relationships between them for policy implications.

**Methods:**

The study employed the baseline category logit models to establish empirical relationships between predictor variables and responses, which reflect different levels of satisfaction.

**Results:**

Income, medical expenditure, and insurance coverage have the positive influence on improving patient satisfaction. However, insurance reimbursement rate has the negative influence. Patients with residency status are more demanding than those without. The more seriously ill, the less likely a patient finds the health services to be satisfactory. The probability of satisfaction conditional on insurance reimbursement is lower for patients with residency status, and higher for those without.

**Conclusion:**

There exist thresholds of income, expenditures, and insurance reimbursement rate, surpassing which probabilistic trends shift. The expenditure threshold for resident patients is almost three times of that for non-residents. An insurance threshold exists only within the group of non-resident patients, ∼65%, suggesting that getting a reimbursement rate higher than this can be very difficult. Therefore, the government’s ambitious goal of universal coverage may be both unrealistic and too rigid as patients with different conditions show different perceptions toward healthcare services.

## Introduction

As a transitional economy, Vietnam’s healthcare system has faced numerous challenges (1) of which providing patients with feasible financing options for medical treatments is one of the thorniest issues. Health insurance is one such option (2, 3). The Vietnamese National Assembly passed an amended Law of Health Insurance in 2014, which has been effective since Jan 2016, stipulating a new set of regulations supposed to reduce poverty risks among local patients by improving health insurance coverage (4).

Although the idea has been welcomed by the populace, it remains to be seen if the actual implementation will meet the public expectation because medical expenditures have increasingly been a problem for a large group of patients (5, 6) while an effective market design for reducing healthcare costs has still been absent (7–9).

In reality, poor people in both urban and rural areas tend to show a low willingness pay for health insurance (10). Unfortunately, this has been one of the main reasons for the risk of destitution among poor patients to significantly increase, causing numerous households to struggle with health shocks, especially in the rural and remote areas (11,12).

Although many scholars advocate the idea that there are possible ways for low-income countries, such as Vietnam, to escape the medical poverty trap (13), the delivery and financing of healthcare services appear to have been more problematic and complicated than most think about (14,15). The situation is in part due to the complication in encouraging health insurance in informal sectors, which are omnipresent in the economy (16), and universal coverage of social health insurance proved to be an elusive target (17).

There has been lack of understanding about how such sociodemographic factors as residency status, the degree of illness, income, insurance and health costs affect trends of patient satisfaction with healthcare services.

This short communication introduces new results obtained from a medical survey in Vietnam in 2015, addressing specific research questions as stated below.

### Research questions

1. Do the continuous variables (income, expenditure, insurance coverage) and categorical variables (residency status, illness) empirically determine patient satisfaction with healthcare service?
2. Do there exist empirical thresholds of income, expenditures and insurance coverage at which patient satisfaction with healthcare services show a probabilistic shift?

The answers to these questions would enhance our understanding and provide evidence for policy-makers in devising policy changes in the future.

## Methods

The survey has been conducted in conformity with strict standards of research ethics, in conformity to: a) The ICMJE Recommendations (Update December 2016); b) The WMA Declaration of Helsinki (Update October 2013); and, c) Decision 460/QD-BYT by the Vietnamese Ministry of Health (February 2012).

Its data collecting and processing practices have met the basic principles of ethical research, namely: (i) Beneficence: As a researcher I strive to ensure that my work makes a positive contribution to the welfare of those affected by it; (ii) Non-malfeasance: I endeavour to ensure that the research work does not cause harm to any sectors of society and, in particular, to participants; (iii) Justice: The benefits and risks associated with this study should be well assessed in advance and both should be equitably distributed throughout society; and, (iv) Autonomy of subjects: The research respects and protects the rights and dignity of participants. The survey was checked by compliance approval numbered V&A/07/2016 by the institutional ethical committee of Vuong & Associates, the survey conducting unit, dated July 15, 2016; then its processes and conducts received the ethical approval number WHUERC-17-07, dated July 13, 2017, by Western University Hanoi’s Research Ethics Committee. The dataset contains 900 records randomly collected from a medical survey on Vietnamese patients conducted in five different provinces in Northern Vietnam–including major cities as Hanoi, Hai Phong, Quang Ninh–from Aug 2014 to Jun 2015. Hospitals from which patients participated in the survey include, but not limited to Viet Duc Hospital, Bach Mai Hospital, Vietnam-Japan Hospital, Hai Duong Polyclinic Hospital, Thai Binh Polyclinic Hospital, Ministry of Transports Polyclinic, to name a few.

The data team consists of people in three main roles: i) data gathering from hospital and insurance agency sources: 03; ii) process coordinating, checking quality and verifying accuracy randomly or if there is some sign of ambiguity: 01; and, iii) putting data into the database: 02. This six-member team approached approximately 3000 patients (or close relatives who answered on behalf of the patients), selected randomly from the hospital records and based on the judgement by data collecting people about whether the patient/relative is available and/or willing to participate, after explaining the ethical standards, issues of information nondisclosure and the possible insights the survey may contribute to the understanding of policy-makers and public in general. Each interview was performed based on a provided questionnaire with the interviewer helping to record the answers. The design of the questionnaire is based on principles of i) statistical standards for categorical data following Agresti’s *Categorical Data Analysis* (18), and continuous data following the World Bank’s reporting for developing countries such as *Health Financing and Delivery in Vietnam*; ii) a literature review of factors that are potentially related, as discussed in the preceding section of Introduction. The questionnaire asks for such key information as their actual medical expenditures, (in) eligibility for insurance coverage, perceived dis/satisfaction about health insurance service, as well as some other such as income, and residency status.

The subset containing data from 605 insured patients is used for analysis, of whom 333 are female and 272 male. Patients’ age spans from 1 to 92, with a majority of 67% belonging to the 40–70 age bracket. The sample size is determined the main rules of modeling categorical data (whether or not with the presence of other continuous data in the specification), satisfying two conditions: i) <20% of cells in the contingency table have count <5; and, ii) no cell with count=0. The sample size is satisfactory (Table 1).

**Table 1:** The rate of responses to health services to be unsatisfactory or not

***Factor***	***Category***	***Obs.***	***Percentage***
“SatServ”	“satis”	206	34.05
	“unsat”	399	65.95
“Res”	“yes”	404	66.78
	“no”	201	33.22
“Ill”	“emerg”	112	18.51
	“bad”	365	60.33
	“light”	128	21.16

Patient satisfaction is a dichotomous response variable (“SatServ”), receiving value of “satis” or “unsat”.

Predictor variables that influence the probability of “SatServ” to take one of the two above values are as follows.

- Residency status (“Res”), with value “yes” if a patient comes from the same region where the healthcare unit is located, and “no” if different;
- Degree of illness (“Ill”) has three categories; “emerg” (hospitalized with an emergency); “bad” (seriously ill), or “light” (moderately or mildly ill);
- Annual income of a patient (“Income”), in millions of Vietnamese Dong (exchange: VND 1 million=US$47);
- Actual treatment expenditures (“Spent”), in millions of Vietnamese Dong;
- Actual insurance reimbursement as a percentage of total expenditure (“Pins”).

The subsequent analysis employs logistic regression, having the specification of Eq.1:

Eq. (1) $$\begin{array}{ll} \text{ln}\left(\frac{\pi (x)}{1-\pi (x)}\right) & =\text{logit}(\pi )\\ & =\beta_{0}+\beta_{i}X_{i}^{K},i\\ & =1,...,K \end{array}$$

In Eq (1), *π*(*x*) represents the success probability, i.e. *Y_i_* = 1; *Y_i_* is the event we want to observe from the empirical data; *β*_0_ is the intercept; and *β_i_* coefficients associated with the *i^th^* predictor variable, *X_i_* · *π*(*x*) is given by: $\pi (x)=\frac{e^{\left(\beta_{0}+\beta_{1}\;X_{1}+...+\beta_{K}\;X_{K}\right)}}{1+e^{\left(\beta_{0}+\beta_{1}\;X_{1}+...+\beta_{K}\;X_{K}\right)}}$ . Actual estimations and technical treatments for the analysis are provided in (18–20). In this study, the success event represents patient satisfaction, that is the response variable in Eq. (1), while *X_i_* are both dichotomous predictor variables of “Res” (residency status) and “Ill” (illness); and continuous variables: “Income”, “Spent”, “Pins”.

## Results

### Descriptive statistics

Table 1 shows that about 66% find the health services to be unsatisfactory. The portion of patients surveyed with a residency is 67%. Approximately 80% of patients report their health status as with an emergency or seriously ill (477/605).

In addition, continuous data given in Table 2 show that the differences among patients are very large as they come from different socioeconomic status (SES) groups, and consume different types of services, lengths of hospitalization.

**Table 2:** Key descriptive statistics for continuous predictor variables employed in BCL models

***Variable***	***Max***	***Min***	***Mean***	***SD***
“Income”	550.00	0.00	42.33	42.65
“Spent”	425.00	1.97	25.42	36.86
“Pins”	0.90	0.00	0.58	0.23

These observations give rise to the need for deeper insights acquired from modeling attempts as presented in the next two results.

### Result for RQ1

The result is provided in Table 3, yielding a set of relations between the response variable “SatServ” and predictor variables “Income”, “Spent”, “Pins”, “Res”, and “Ill”.

**Table 3:** Estimation results for RQ1

	***Intercep***	***“Income”***	***“Spent”***	***“Pins”***	***“Res”***	***“Ill”***	
	**t**				“yes”	“emerg”	“light”
Logit(satis|unsat)	*β*_0_	*β*_1_	*β*_2_	*β*_3_	*β*_4_	*β*_5_	*β*_6_
	0.172	0.017^***^	0.027^***^	−2.658^***^	−1.521^***^	−0.225	0.604^*^
	[0.397]	[3.906]	[4.871]	[−4.797]	[−5.237]	[−0.686]	[2.069]

Signif. codes: 0 ‘^***^’ 0.001 ‘^**^’ 0.01 ‘^*^’ 0.05 ‘.’ 0.1 ‘ ’ 1, z-value in square brackets; baseline category for: “Res”=“no”; “Ill”=“bad”. Residual deviance: 497.57 on 598 degrees of freedom.

Most coefficients are highly significant, indicating plausible relations between variables in consideration.

Empirical probabilities for patient satisfaction conditional on values of predictor variables can be computed. For instance, for a patient with residency status, with an annual income of VND 100 million (US$4,700), being seriously ill, paying VND 40 million for treatment expenditures, and with insurance coverage of 50%, the probability that patient finds the services to be satisfactory is 52.5%, computed as follows:

$$\pi_{\mathrm{satis}}=\frac{{\text{e}}^{\left(0.172+0.017\times 100+0.027\times 40-2.658\times 0.5-1.521\right)}}{1+{\text{e}}^{\left(0.172+0.017\times 100+0.027\times 40-2.658\times 0.5-1.521\right)}}=0.525.$$

### Result for RQ2

This set provides estimations for “SatServ” and predictor variables “Res”, “Ill” and one of the three continuous variables “Income”, “Spent”, “Pins”, for each result as reported in Table 4.

**Table 4:** Three estimation results for RQ2

	***Intercept***	***“Income”***	***“Res”***	***“Ill”***	
			“yes”	“emerg”	“light”
Logit(Satis|Unsat)	*β*_0_	*β*_1_	*β*_2_	*β*_3_	*β*_4_
	0.143 [0.687]	0.021^***^ [4.739]	−2.862^***^ [−12.146]	−0.233 [−0.800]	0.556^*^ [2.018]
		Estimation 4(A)			
	Intercept	“Spent”	“Res”	“Ill”	
			“yes”	“emerg”	“light”
Logit(Satis|Unsat)	*β*_0_	*β*_1_	*β*_2_	*β*_3_	*β*_4_
	−0.643^*^ [−2.274]	0.030^***^ [5.632]	−1.690^***^ [−6.645]	−0.475 [−1.515]	1.257^***^ [4.879]
		Estimation 4(B)			
	Intercept	“Pins”	“Res”	“Ill”	
			“yes”	“emerg”	“light”
Logit(Satis|Unsat)	*β*_0_	*β*_1_	*β*_2_	*β*_3_	*β*_4_
	2.190^***^ [6.986]	−3.057^***^ [−5.925]	−2.073^***^ [−9.156]	−0.199 [−0.671]	0.550^***^ [2.055]

Estimation 4(c)

Sig. codes: 0 ‘^***^’ 0.001 ‘^**^’ 0.01 ‘^*^’ 0.05 ‘.’ 0.1 ‘ ’ 1, z-value in square brackets; baseline category for: “Res”=“no”; “Ill”=“bad”. Residual deviance: 556.741 on 600 degrees of freedom.

Table 4 enables the computing of “thresholds” and an example follows. Subtable 4(a) has a functional form of Eq.(RQ2.1):

Eq. (RQ2.1) $$\begin{array}{ll} \text{ln}\left(\frac{\pi_{\mathrm{satis}}}{\pi_{\mathrm{unsat}}}\right) & =0.143+0.021\times \mathrm{Income}\\ & \;-2.862\times \mathrm{yesRes}-0.233\\ & \;\times \mathrm{EmergIll}+0.556\\ & \;\times \mathrm{LightIll} \end{array}$$

Thus, a probability of patient satisfaction conditional upon “Res”, “Ill”, and “Income” is:

$$\pi_{\mathrm{satis}}=\frac{{\text{e}}^{\left(0.143+0.021\times \mathrm{Income}-2.862\times \mathrm{yesRes}-0.233\times \mathrm{EmergIll}+0.556\times \mathrm{LightIll}\right)}}{1+{\text{e}}^{\left(0.143+0.021\times \mathrm{Income}-2.862\times \mathrm{yesRes}-0.233\times \mathrm{EmergIll}+0.556\times \mathrm{LightIll}\right)}}$$

For each value of “Res”, “Ill” we can attempt to determine numerical value of “threshold income”. For instance, for “Res”=“yes”; “Ill”=“emerg”, then:

$$\pi_{\mathrm{satis}}=\frac{{\text{e}}^{\left(-2.952+0.021\times \mathrm{Income}\right)}}{1+{\text{e}}^{\left(-2.952+0.021\times \mathrm{Income}\right)}}$$

**Fig. 1:**
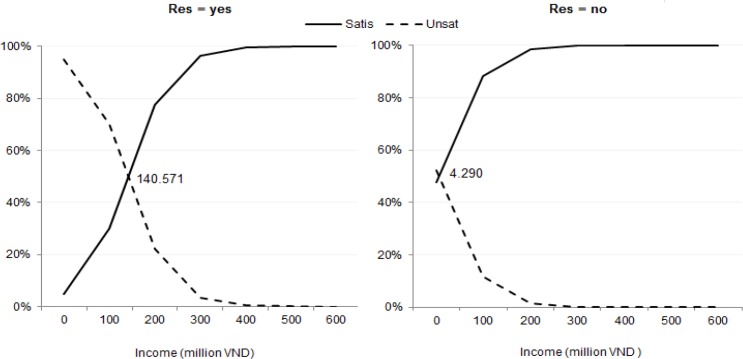
Probabilities of patient dis/satisfaction for patients with an emergency, conditional on income

In our definition, “threshold income” is the level of income at which *π_satis_* = 50% ; thus the computed “threshold income” in this situation is VND 140.6 million (US$6,600). In the same vein, the income threshold for “Res”=“no” (“Ill” remains “emerg”) is VND 4.29 million. These two thresholds are presented in Fig. 1.

In the same vein, many more thresholds for different conditions can be computed and the changing patterns of conditional probabilities of dis/satisfaction can be observed.

## Discussion

The empirical results indicate that both income and actual expenditure have the positive influence on improving patient satisfaction. However, the influence of insurance reimbursement rate is negative *β*_3_= −2.658 (*P*<0.0001). A possible explanation is that the time and effort or even money (as corruption is not uncommon at hospitals) may make most patients think: “It is not worth spending the time and making effort to have a unit of increase in insurance benefits”. Furthermore, it is more difficult to satisfy patients coming from the same region as the healthcare unit; *β*_4_= −1.521 (*P*<0.0001). Finally, as β_5_<0 and *β*_6_>0, the more seriously ill, the fewer patients find the health services to be satisfactory. The next implication is about some of the thresholds. The probability of satisfaction conditional on insurance reimbursement is lower for patients with residency status, in the range of 4.1% to 66%; but for patients without residency (“Res”=“no”) from 25.6% to 93.9%.

**Fig. 2:**
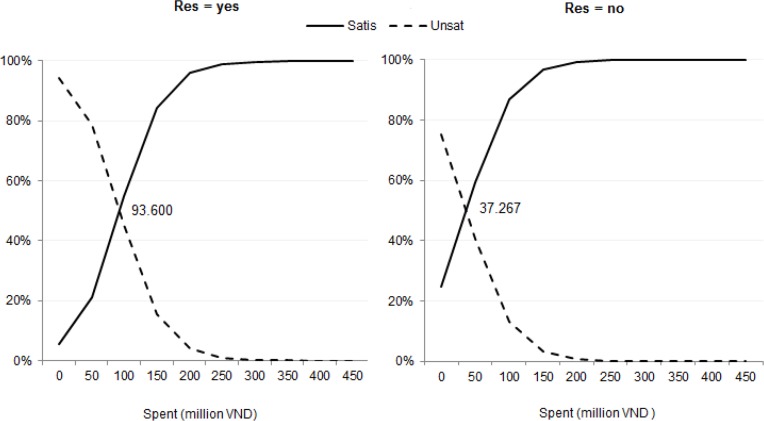
Probabilities of patient dis/satisfaction for patients with an emergency, conditional on medical expenditure

For “expenditure threshold” in Fig. 2 the threshold jumps from VND 37.3 million (US$1,750) for non-resident patients to VND 93.6 million (US$4,400).

**Fig. 3:**
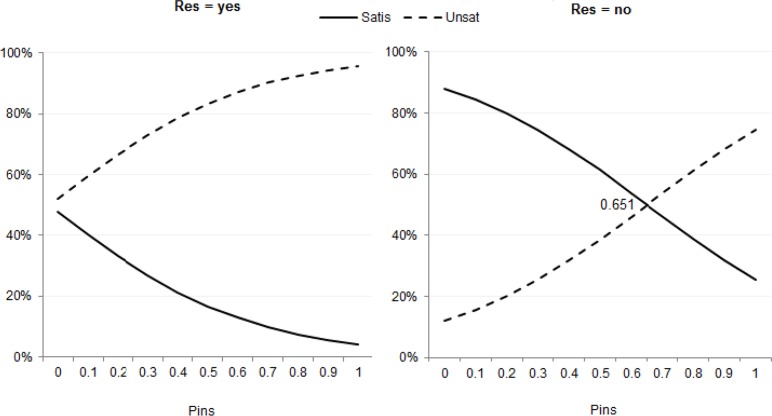
Probabilities of patient dis/satisfaction for patients with an emergency, conditional on medical insurance coverage

In addition, Fig. 3 suggests that “insurance threshold” only exists among non-resident patients, ∼65%. This insight is counter-intuitive, as most believe that the higher insurance reimbursement rate is the happier a patient becomes.

## Conclusion

Firstly, Vietnamese government’s ambitious goal of universal coverage may be both unrealistic and too rigid as patients with varying sociodemographic conditions show different perceptions toward healthcare services and influences of factors. In fact, a reimbursement rate of >65% has empirically been very difficult; and aiming for a higher rate might incur more costs than the benefits patients receive. Secondly, in a low-resource setting in transition like Vietnam, the computed thresholds are meaningful as they make evidence-based policy making possible and efficient, such as targeting the right group for spillover effects of insurance benefits: non-resident poor patients.

## Ethical considerations

Ethical issues, including plagiarism, informed consent, misconduct, data fabrication and/or falsification, double publication and/or submission, redundancy, etc., have been completely observed by the author.

## References

[1] Ekman B Nguyen TL Ha AD Axelson H (2008). Health insurance reform in Vietnam: a review of recent developments and future challenges. Health Policy Plan, 23(4): 252–263. 18424793 10.1093/heapol/czn009

[2] Ensor T (1995). Introducing health insurance in Vietnam. Health Policy Plan, 10(2): 154–163. 10143453 10.1093/heapol/10.2.154

[3] Habib SS Perveen S Khuwaja HMA (2016). The role of micro health insurance in providing financial risk protection in developing countries-a systematic review. BMC Public Health, 16: 281. 27004824 10.1186/s12889-016-2937-9 PMC4802630

[4] Vietnamese National Assembly (2014). Law No. 46/2014/QH13: Amendments to the Law on Health Insurance. http://www.iexpertvn.com/2015/01/law-no-462014qh13-amendments-to-lawon.html

[5] Vuong QH (2015). Be rich or don’t be sick: estimating Vietnamese patients’ risk of falling into destitution. Springerplus, 4: 529. 26413435 10.1186/s40064-015-1279-x PMC4577521

[6] Cramton P Katzman B (2010). Reducing healthcare costs requires good market design. http://www.cramton.umd.edu/papers2010-2014/cramton-katzman-reducing-healthcare-costs.pdf

[7] Hoang VM Oh J Tran TA (2015). Patterns of health expenditures and financial protections in Vietnam 1992–2012. J Korean Med Sci, 30(Suppl 2): S134–8. 26617446 10.3346/jkms.2015.30.S2.S134 PMC4659865

[8] Jowett M Contoyannis P Vinh ND (2003). The impact of public voluntary health insurance on private health expenditures in Vietnam. Soc Sci Med, 56(2): 333–342. 12473318 10.1016/s0277-9536(02)00031-x

[9] Lagomarsino G Garabrant A Adyas A (2012). Moving towards universal health coverage: health insurance reforms in nine developing countries in Africa and Asia. Lancet, 380(9845): 933–943. 22959390 10.1016/S0140-6736(12)61147-7

[10] Lofgren C Thanh NX Chuc NT (2008). People’s willingness to pay for health insurance in rural Vietnam. Cost Eff Resour Alloc, 6: 16. 18691440 10.1186/1478-7547-6-16 PMC2527552

[11] Mitra S Palmer M Mont D Groce N (2016). Can households cope with health shocks in Vietnam? Health Econ, 25(7): 888–907. 26017577 10.1002/hec.3196 PMC4975721

[12] Nguyen C (2016). The impact of health insurance programs for children: evidence from Vietnam. Health Econ Rev, 6: 34. 27491776 10.1186/s13561-016-0111-9 PMC4974211

[13] Whitehead M Dahlgren G Evans T (2001). Equity and health sector reforms: can low-income countries escape the medical poverty trap? Lancet, 358(9284): 833–836. 11564510 10.1016/S0140-6736(01)05975-X

[14] Wagstaff A Lieberman SS (2009). Health financing and delivery in Vietnam. World Bank: Washington D.C.; http://documents.worldbank.org/curated/en/295021468124767254/pdf/473880PUB0VN0H101OFFICIAL0USE0ONLY1.pdf

[15] Tran VT Hoang TP Mathauer I Nguyen TKP (2011). A health financing review of Vietnam with a focus on social health insurance. World Health Organization: Geneva http://www.who.int/health_financing/documents/cov-oasis_e_11-vietnam/en/

[16] Wagstaff A Nguyen HTH Dao H Bales S (2016). Encouraging health insurance for the informal sector: a cluster randomized experiment in Vietnam. Health Econ, 25(6): 663–674. 26666771 10.1002/hec.3293

[17] Somanathan A Tandon A Dao HL (2014). Moving toward universal coverage of social health insurance in Vietnam: assessment and options. World Bank: Washington D.C.; http://documents.worldbank.org/curated/en/383151468138892428/pdf/890660PUB0Univ00Box385269B00PUBLIC0.pdf

[18] Agresti A (2013). Categorical Data Analysis. 3rd ed Hoboken: Wiley. New Jersey.

[19] Penn State Science Analysis of discrete data: further topic on logistic regression. https://onlinecourses.science.psu.edu/stat504/node/217

[20] Vuong QH (2017). Survey data on Vietnamese propensity to attend periodic general health examinations. Sci Data, 4: 2017142 https://www.nature.com/articles/sdata2017142. 10.1038/sdata.2017.142 PMC5625553 28972572

